# The complete chloroplast genome of *Agrostis capillaris* L

**DOI:** 10.1080/23802359.2021.1959446

**Published:** 2021-07-28

**Authors:** Hongyan He, Jiayi Fu, Qianqian Wang, Qianqian Xi, Xiangyu Wei, Yi Zhao, Chen Ling, Tianliang Chang, Yuwei Zhao

**Affiliations:** a Provincial Key Laboratory of Biotechnology of Shaanxi Province, Northwest University, Xi’an, China; b College of Life Sciences, Northwest University, Xi’an, China; c Key Laboratory of Resource Biology and Biotechnology in Western China (Ministry of Education), Northwest University, Xi’an, China

**Keywords:** *Agrostis capillaris*, chloroplast genome, phylogenetic analysis

## Abstract

*Agrostis capillaris* is a cool-season turf grass species that is found worldwide in temperate countries, and a good Pb phytostabilizer. In this study, the entire chloroplast genome sequence of *A. capillaris* was determined by Illumina sequencing. The complete chloroplast genome was circular and composed of 136,396 bp nucleotides with a GC content of about 38.5%. There were a large single-copy region (LSC, 81,659bp), a small single-copy region (SSC, 12,593bp), and a pair of reverse repeat regions (IRs, 42,144bp) in the chloroplast genome. In total, the *A. capillaris* chloroplast genome contained 144 genes, including 96 protein-coding genes, 40 tRNA genes, and 8 rRNA genes. Phylogenetic analysis revealed that *A. capillaris* was closely related to *A. gigantean*. The sequence data of *A. capillaris* chloroplast genome could provide useful genetic information for the studies on phylogenetic and evolutionary of Agrostidinae.

*Agrostis capillaris* is classified as a cool-season turf grass species in the Gramineae family, and has been used for landscaping widespread. This grass is found worldwide in temperate countries (Saarela et al. 2017). *A. capillaris* can tolerate high concentrations of Pb and is a good Pb phytostabilizer (Lebrun et al. 2021). And Pb concentrations in *Agrostis* plants are higher in the roots compared to the aerial parts (Rodríguez-Seijo et al. 2016). The Pb phytostabilization capacity of *A. capillaris* has be revealed through the considerable accumulation of Pb in roots and the presence of secondary mineral in the soil (Dahmani-Muller et al. 2000). This secondary mineral interacts with Pb and reduces its mobility, eliminating it from the metabolisms of plants (Patricia and Fernandez-Cirelli 2008). However, there are few reports about the chloroplast genome analysis of *A. capillaris*. In this study, we reported the complete chloroplast genome of *A. capillaris*, which will provide useful genetic information for the studies on phylogenetic and evolutionary of Agrostidinae.

Fresh leaves of *A. capillaris* were collected from three different plantlets at the Botanical Garden of Northwest University (34°25'N, 108°93'E). Those leaves were stored at the Northwest University herbarium (Yuwei Zhao, zhaoyw@nwu.edu.cn) under the voucher number 2021102. The total genomic DNA was isolated from those leaves by a modified CTAB method (Doyle 1987). The specific modification was that five volumes (about 500 μL) ice-cold absolute ethanol were used to precipitate DNA overnight at −20 °C. The DNA samples were deposited at −80 °C at the Provincial Key Laboratory of Biotechnology of Shaanxi Province, Xi’an, China. The complete chloroplast genome of *A. capillaris* was sequenced by Illumina NovaSeq PE150 System at Beijing Biomarker Technologies Co., Ltd., and assembled through GetOrganelle program (Jin et al. 2018) with reference to *Oryza sativa* chloroplast genome (NC_031333). The complete chloroplast genome of *A. capillaris* was annotated in Geneious ver. 8.0.2, and then submitted to GenBank (Accession No. MW143240).

The *A. capillaris* circular chloroplast genome was 136,396 bp and contained a large single-copy region (LSC) of 81,659 bp, a small single-copy region (SSC) of 12,593 bp and a pair of reverse repeat regions (IRs) of 42,144 bp.

There were 144 genes in the chloroplast genome, including 96 protein-coding genes, 40 tRNA genes, and 8 rRNA genes. The overall GC content of the chloroplast genome was about 38.5%.

To determine the phylogenetic position of *A. capillaris* within the family Gramineae, the complete chloroplast genome sequencs of 16 Gramineae species and two Asteraceae species were aligned with MAFFT version 7.450 (Katoh and Standley 2013) and then visualized and manually adjusted using BioEdit (Hall 1999). A maximum likelihood (ML) tree was performed using MEGA 7.0 (Sudhir et al. 2016) with 1000 bootstrap replicates. The result indicated that *A. capillaris* was sister to *A. gigantean* (Figure 1).

**Figure 1.**
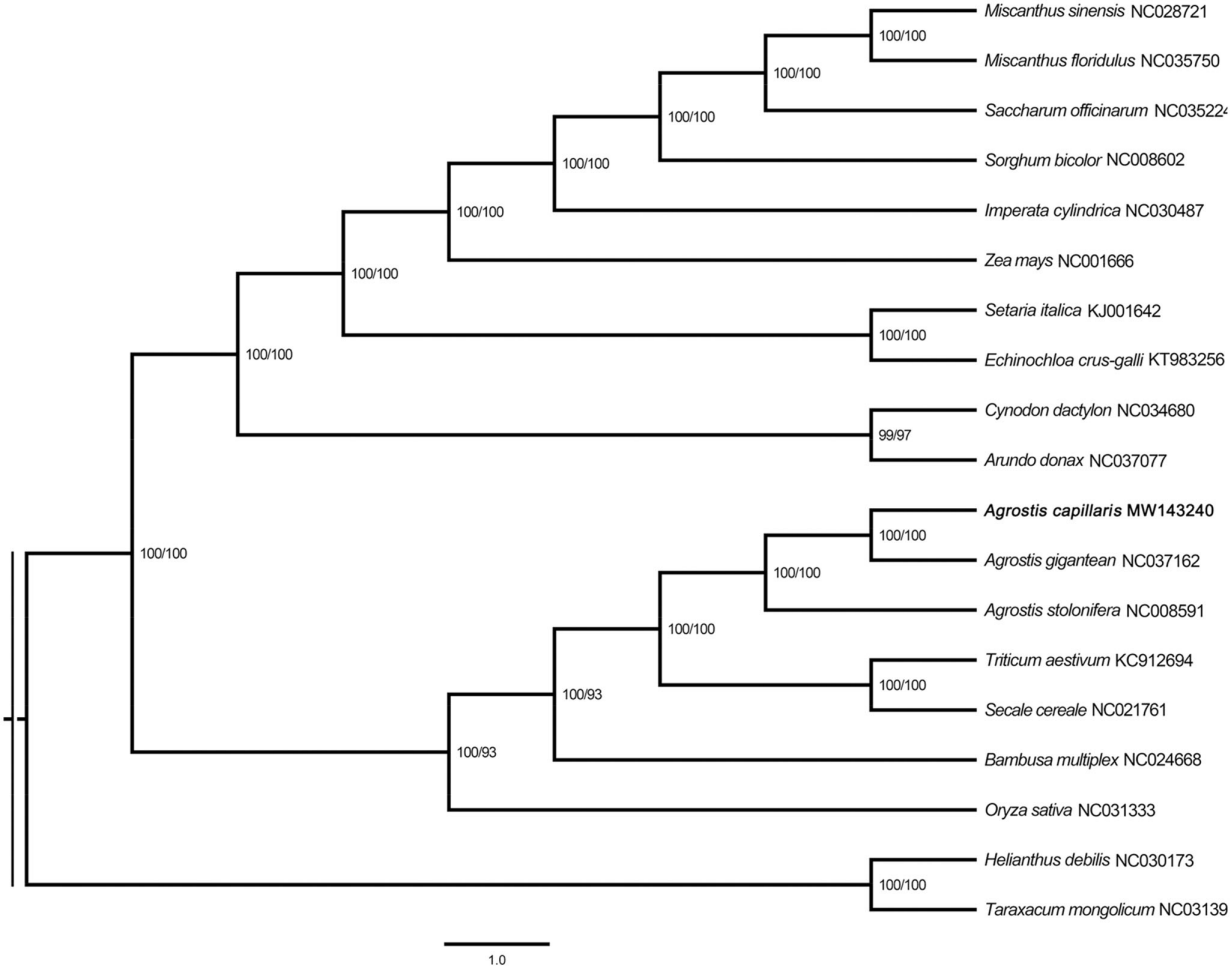
Maximum likelihood (ML) tree based on the complete chloroplast genome sequences from eighteen species.

## Data Availability

The genome sequence data that support the findings of this study are openly available in GenBank of NCBI at (https://www.ncbi.nlm.nih.gov/) under the accession number: MW143240. The associated BioProject, SRA, and Bio-Sample numbers are PRJNA698087, SRR13617003 and SAMN17705311, respectively.
